# Contrast-enhanced ultrasound manifestations of renal masses undetectable on conventional ultrasound

**DOI:** 10.3389/fonc.2022.943960

**Published:** 2022-07-25

**Authors:** Lingling Tao, Jinfang Fan, Weiwei Zhan, Weiwei Li, Jian Lu, Nanan Yang, Binbin Ma, Wei Zhou

**Affiliations:** ^1^ Department of Ultrasound, Ruijin Hospital LuWan Branch, Shanghai Jiaotong University School of Medicine, Shanghai, China; ^2^ Department of Ultrasound, Ruijin Hospital, Shanghai Jiaotong University School of Medicine, Shanghai, China; ^3^ Department of Interventional Radiology, Ruijin Hospital LuWan Branch, Shanghai Jiaotong University School of Medicine, Shanghai, China; ^4^ Department of Urology, Ruijin Hospital LuWan Branch, Shanghai Jiaotong University School of Medicine, Shanghai, China

**Keywords:** conventional ultrasound, non-enhanced computed tomography, renal mass, undetectable, contrast-enhanced ultrasound, clear cell renal cell carcinoma, urothelial carcinoma of the renal pelvis

## Abstract

This study aimed to retrospectively analyze the features of contrast-enhanced ultrasound (CEUS) of renal masses that cannot be detected by conventional ultrasound (CUS). The data of 264 patients who underwent CEUS for renal lesions from January 2016 to December 2019 were retrieved. Of these, 16 patients with renal masses which were not detected by CUS were included in the final analysis. The corresponding characteristics of CEUS were evaluated, including intensity of enhancement, homogeneity, wash-in and wash-out patterns, and perilesional rim-like enhancement. Of the 16 patients, 10 patients had clear cell renal cell carcinoma (ccRCC) and 6 patients had urothelial carcinoma of the renal pelvis (UCRP). Compared with the location on non-enhanced computed tomography (CT) scan, all tumors were detected on CEUS. Most (7/10) of the ccRCCs appeared as hyperenhancement, homogeneous enhancement, synchronous-in, and no perilesional rim-like enhancement. Most (4/6) of the UCRPs appeared as isoenhancement, slow-in, fast-out, and no perilesional rim-like enhancement. CEUS may be helpful in the diagnosis and differential diagnosis of renal tumors which were not observed on CUS, and it might be an alternative method for some patients when contrast-enhanced computed tomography (CECT) or magnetic resonance imaging (MRI) cannot be performed.

## Introduction

The differential diagnosis of renal tumor histotypes is vital for clinical treatment decision-making and prognosis evaluation. Imaging examination is the main basis for clinical differentiation of renal tumor histotypes, which is of great significance (1). Most of the patients with renal masses are asymptomatic in the early stage, and 70%–80% of renal tumors can be detected by ultrasound (US) in routine physical examination (2). Although conventional ultrasound (CUS), including B mode and color Doppler, has an important role in the diagnosis of renal tumors, it also has some limitations. Factors such as obesity, growth pattern, echo, and location may interfere with the CUS examination, leading to misdiagnosis or missed diagnosis, which often requires further examinations (3, 4).

Contrast-enhanced ultrasound (CEUS) was recently introduced as a promising technique for the evaluation of renal tumors (5). CEUS is performed by using a microbubble contrast agent. As the size of the microbubbles is similar to red cells (ranging from 1 to 10 µm), the microbubbles remain completely in the intravascular space with no nephrotoxicity and discharge through the respiratory system. The European Federation of Societies for Ultrasound in Medicine and Biology Guidelines and Recommendations on the Clinical Practice of CEUS have suggested indications for CEUS of renal diseases (6). CEUS is helpful in evaluating atypical cysts and uncertain masses detected by computed tomography (CT) or magnetic resonance imaging (MRI) (7). In addition to the role in the differential diagnosis of renal masses, CEUS can also detect masses that cannot be observed on CUS; however, there were very few related studies (8). In our daily work, we also found some cases with renal masses which were not detected by CUS. These patients had a single suspicious renal mass on non-enhanced CT scan. However, they were unable to undergo contrast-enhanced computed tomography (CECT) or MRI for their own reasons, so CEUS was then performed at the ultrasound department, which clearly showed the masses. This study aimed to analyze CEUS features of renal masses that were undetectable by CUS.

## Materials and methods

### Patients

This retrospective study was approved and supervised by the institutional review committee of our hospital, and informed consent was obtained from each patient prior to the CEUS examination. The data of 264 patients from January 2017 to December 2020 were retrieved. Of these, 16 patients with renal masses were included in the final analysis. Histopathological evaluation was performed on the specimens obtained from surgically resected lesions. The inclusion criteria were as follows: 1) no renal mass was found on CUS; 2) a single suspicious renal mass was observed on CT; 3) CEUS was performed after CT examination; and 4) patients had not undergone any invasive treatments before. The exclusion criteria were as follows: 1) a renal mass that can be easily identified by CUS; 2) cases that were confirmed by CECT or MRI; 3) cases who had incomplete imaging data; and 4) patients who were pregnant, had a history of cardiac failure, or had respiratory disorders.

### Imaging technology and technical characteristics

US examination was performed by using an ultrasonic diagnostic instrument (Aplio 500, Canon Medical Systems, Tokyo, Japan), equipped with a probe 6C1 (frequency of 3.5–5.0 MHz) for CUS and CEUS with a mechanical index of 0.07. Compared with the location of the renal mass shown on the non-enhanced CT scan, CUS was used to identify the renal mass. At the same time, CUS was used to observe whether the renal pelvis was separated. If the mass was still not found, CEUS was performed on the area suspected by non-enhanced CT scan.

The area suspected by non-enhanced CT scan was then targeted, and imaging settings such as depth, gain, and focal zone were optimized to ensure adequate image quality. CEUS was administered by injecting 1.2–2.4 ml of SonoVue (Bracco, Italy) through an antecubital vein followed by flushing with 5.0 ml of normal saline. All dynamic images were observed for 3 min and stored on a hard disk for further analysis. All CEUS examinations were evaluated by two radiologists with more than 10 years of experience in CEUS. Differences in opinions and findings were discussed and resolved by the same two radiologists.

### Analysis of CEUS

The enhancement pattern and characteristics of CEUS were evaluated according to the literature (9). A) Enhancement intensity at peak: the enhancement degree of the lesion was compared with that of the renal cortex, and it was classified as hyper-, iso-, or hypoenhancement. B) Enhanced homogeneity: the homogeneity was divided into homogeneous and heterogeneous. C) Wash-in pattern was classified as “fast-in,” “synchronous-in,” or “slow-in,” indicating that the contrast agent entered the mass faster than, the same as, and more slowly than the adjacent renal cortex, respectively. D) Wash-out pattern was divided into “fast-out,” “synchronous-out,” or “slow-out,” indicating that the contrast agent discharged from the mass faster than, the same as, and more slowly than the adjacent cortex, respectively. E) Perilesional rim-like enhancement was divided into present or absent.

### Analysis of CUS after CEUS

In the simultaneous display mode of both CUS and CEUS images, the mass displayed on CEUS was delineated, and the corresponding position of the mass on CUS was also automatically delineated. The CUS features of the suspicious area were analyzed. CUS features included echogenicity, homogeneity, and blood flow signal. The echogenicity was classified as hypoechoic, isoechoic, or hyperechoic when compared with that of the adjacent renal cortex. Homogeneity was classified as homogeneous and heterogeneous. The blood flow signal inside the tumor was divided into yes or no.

## Results

A total of 6 women and 10 men were recruited, with a mean age of 62.2 ± 12.2 years (range, 38–83 years). There were a total of 16 masses, with a mean maximum diameter of 2.2 ± 0.7 cm (range, 1.2–3.8 cm). Of the 16 tumors, 9 (56.3%) were on the left, and the remaining 7 (43.7%) were on the right. All the masses were diagnosed by postoperative pathology. The pathological results were clear cell renal cell carcinoma (ccRCC) in 10 patients (62.5%), with a mean maximum diameter of 2.0 ± 0.4 cm (range, 1.5–2.9 cm), and urothelial carcinoma of the renal pelvis (UCRP) in 6 patients (37.5%), with a mean maximum diameter of 2.6 ± 0.9 cm (range, 1.2–3.8 cm) (**Table 1**).

**Table 1:** Clinical characteristics of the 16 patients with renal masses.

Case	Gender	Age	Location	Size (cm)	Clinical presentation	Reasons why CECT or MRI was not performed	Surgical methods	Pathology
1	Female	83	Right	1.9 × 1.6	Asymptomatic	Chronic renal failure	PN	ccRCC
2	Female	52	Left	1.5 × 1.3	Pain and discomfort in the left waist	Allergy to contrast media	PN	ccRCC
3	Male	38	Left	1.8 × 1.6	Asymptomatic	Allergy to contrast media	PN	ccRCC
4	Male	43	Right	1.6 × 1.5	Asymptomatic	Unwilling	PN	ccRCC
5	Male	71	Right	2.1 × 1.9	Pain and discomfort in the right waist	Allergy to contrast media	PN	ccRCC
6	Male	69	Left	2.9 × 2.1	Asymptomatic	Unwilling	RN	ccRCC
7	Female	57	Right	2.7 × 1.8	Asymptomatic	Unwilling	RN	ccRCC
8	Male	65	Right	1.5 × 1.2	Pain and discomfort in the right waist	Renal dysfunction	PN	ccRCC
9	Male	58	Left	1.7 × 1.6	Asymptomatic	Unwilling	PN	ccRCC
10	Male	71	Left	2.3 × 2.1	Asymptomatic	Unwilling	PN	ccRCC
11	Female	56	Right	2.4 × 1.8	Pain and discomfort in the right waist, gross hematuria	Allergy to contrast media	RN with excision of bladder cuff	UCRP
12	Female	69	Right	2.2 × 1.6	Gross hematuria	Renal dysfunction	RN	UCRP
13	Male	66	Left	1.2 × 7.8	Pain and discomfort in the left waist	Allergy to contrast media	RN with excision of bladder cuff	UCRP
14	Male	74	Left	3.8 × 3.3	Pain and discomfort in the left waist, gross hematuria	Unwilling	RN with excision of bladder cuff	UCRP
15	Male	73	Left	3.4 × 1.9	Gross hematuria	Renal dysfunction	RN	UCRP
16	Male	50	Left	2.4 × 2.1	Gross hematuria	Allergy to contrast media	RN	UCRP

ccRCC, clear cell renal cell carcinoma; UCRP, urothelial carcinoma of the renal pelvis; RN, radical nephrectomy; PN, partial nephrectomy.

**Figure 1:**
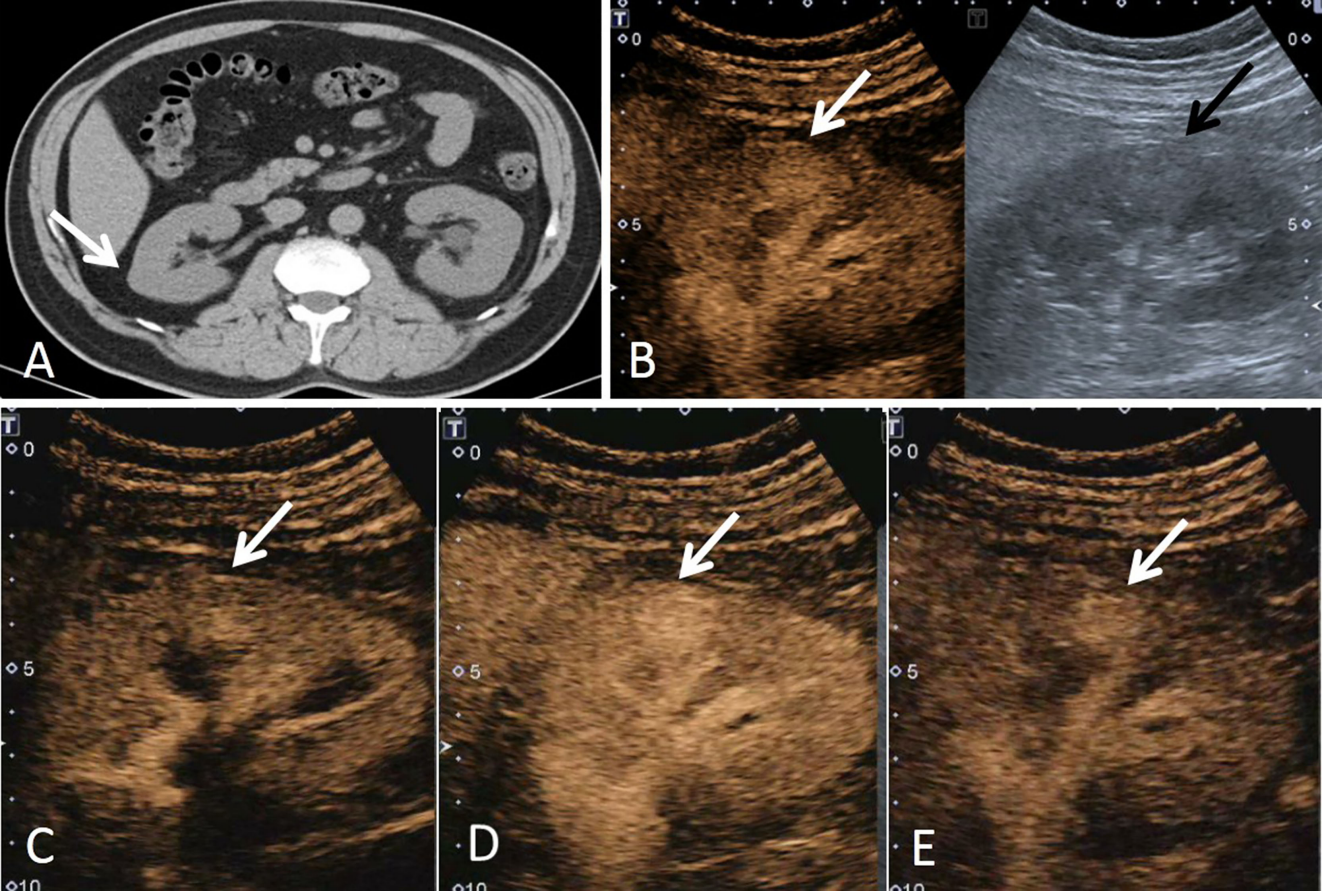
A 43-year-old man had a ccRCC in the right kidney with the size of 1.6 × 1.5 cm. **(A)** Non-enhanced CT showed a suspicious lesion in the middle of the right kidney (white arrow). **(B)** According to the position displayed on CT, the simultaneous display mode of conventional ultrasound (CUS, right) and contrast-enhanced ultrasound (CEUS, left) was performed, and CEUS showed a lesion with hyperenhancement (white arrow), but it was not detected on CUS (black arrow). **(C)** On CEUS imaging, it showed that the contrast agent entered the mass synchronously with the adjacent renal cortex (white arrow). **(D)** At the peak of enhancement intensity, it showed homogeneous hyperenhancement compared with that of the renal cortex (white arrow). **(E)** When the contrast agent discharged from the mass, it showed a slow-out pattern compared to the adjacent cortex (white arrow).

**Figure 2:**
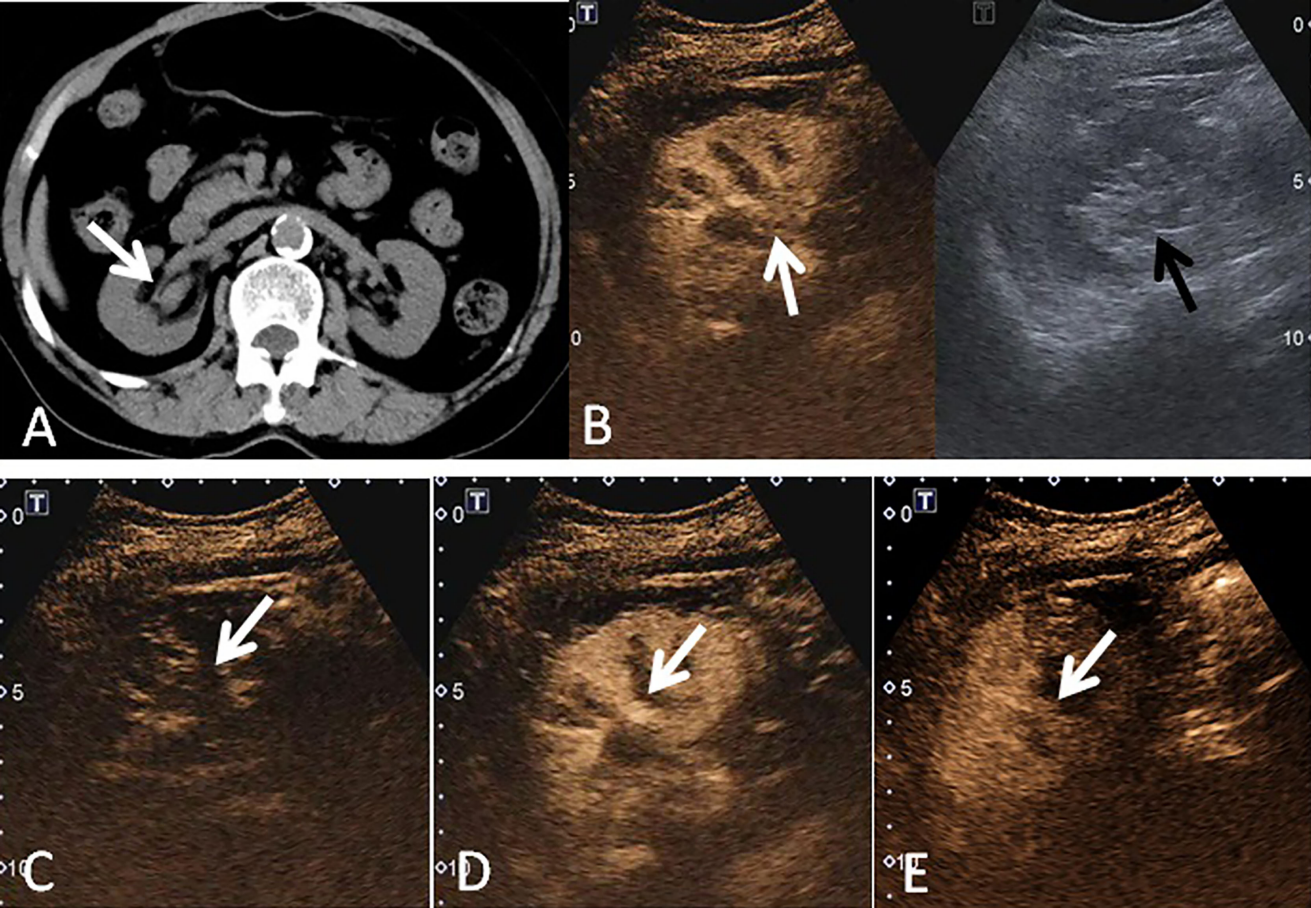
A 69-year-old woman had a UCRP in the right kidney with the size of 2.2 × 1.6 cm. **(A)** A suspicious renal pelvis mass (white arrow) was observed on non-enhanced CT scan. **(B)** According to the position displayed on CT, the simultaneous display mode of CUS (right) and CEUS (left) was performed, and CEUS demonstrated local contrast enhancement in the renal pelvis (white arrow). CUS showed a slight separation of the renal pelvis, but no lesion was observed (black arrow). **(C)** On CEUS imaging, it showed a slow-in pattern compared to the renal cortex (white arrow). **(D)** The enhancement intensity of the tumor was similar to that of the adjacent renal cortex (white arrow). **(E)** Compared to the renal cortex, the tumor showed a fast-out pattern (white arrow) on CEUS.

**Figure 3:**
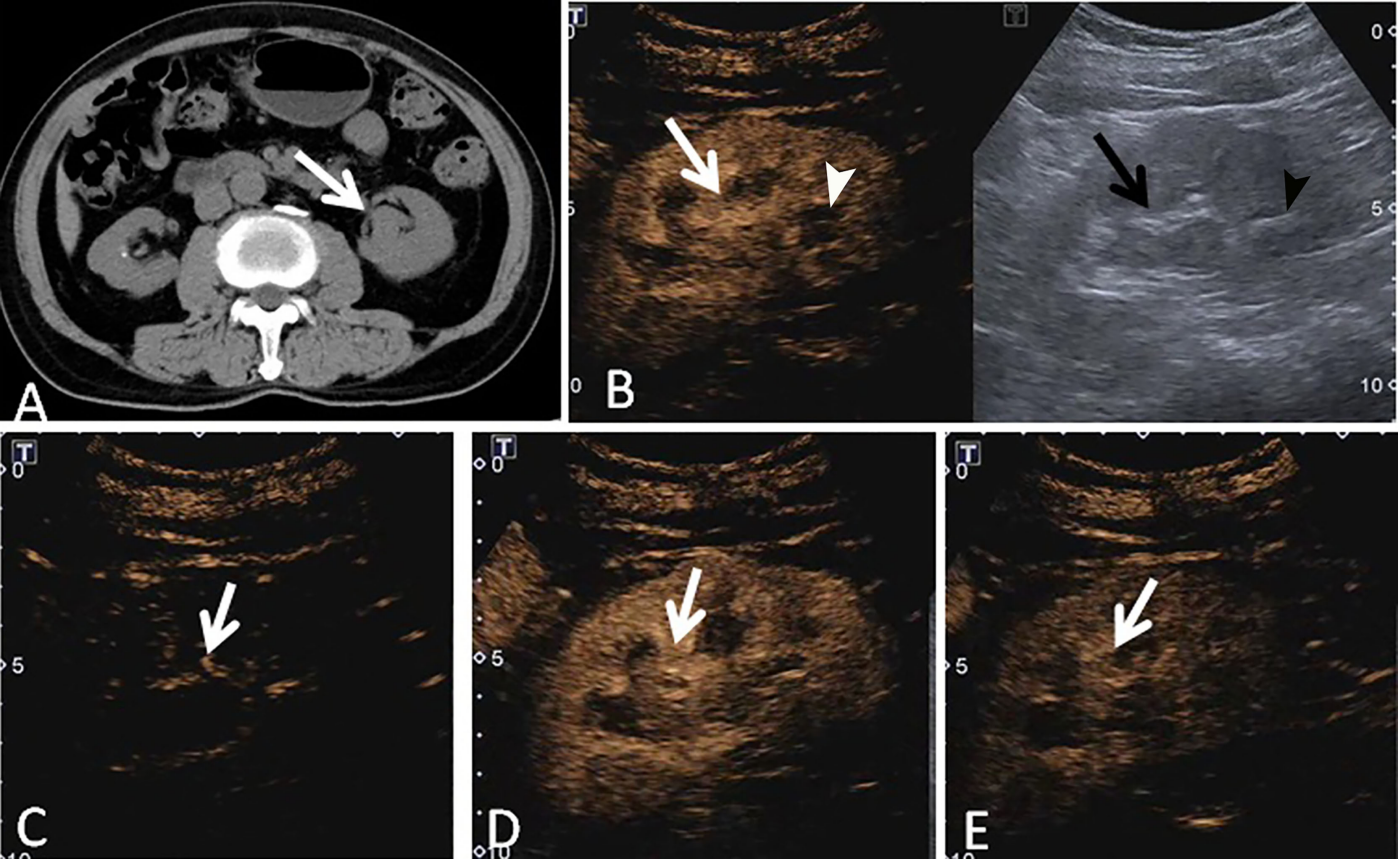
A 73-year-old man had a UCRP in the left kidney with the size of 3.4 × 1.9 cm. **(A)** Non-enhanced CT scan showed that there may be blood clots in the renal pelvis (white arrow). **(B)** According to the position displayed on CT, the simultaneous display mode of CUS (right) and CEUS (left) was performed. No obvious lesion (black arrow) was detected on CUS, but it showed a slight separation of the renal pelvis and a stone (black arrowhead) in the lower part of the renal pelvis. On CEUS, a lesion with enhancement was observed (white arrow), and the stone showed no enhancement (white arrowhead). **(C)** The contrast agent entered the lesion more slowly than that of the adjacent renal cortex (white arrow). **(D)** The tumor showed heterogeneous enhancement, and the enhancement intensity of the tumor (white arrow) was similar to that of the adjacent renal cortex. **(E)** The contrast agent discharged from the mass quickly, and the tumor (white arrow) showed a fast-out pattern compared to the renal cortex.

Of the 10 cases with ccRCC, 10 (100.0%) showed hyperenhancement on CEUS. Six cases (60.0%) showed homogeneous enhancement, and seven cases (70.0%) showed a synchronous-in pattern (**Figure 1**). Of the six patients with UCRP, five (83.3%) showed a slow-in pattern, four (66.7%) showed a fast-out pattern, and the enhancement intensity was isoenhancement in four cases (66.7%) (**Figure 2**). In one case of UCRP with renal pelvis stones, CEUS showed an isoenhancement lesion next to the stones, while it was suspicious for a thrombus or a mass on CT (**Figure 3**). Perilesional rim-like enhancement was not observed in all the cases.

**Figure 4:**
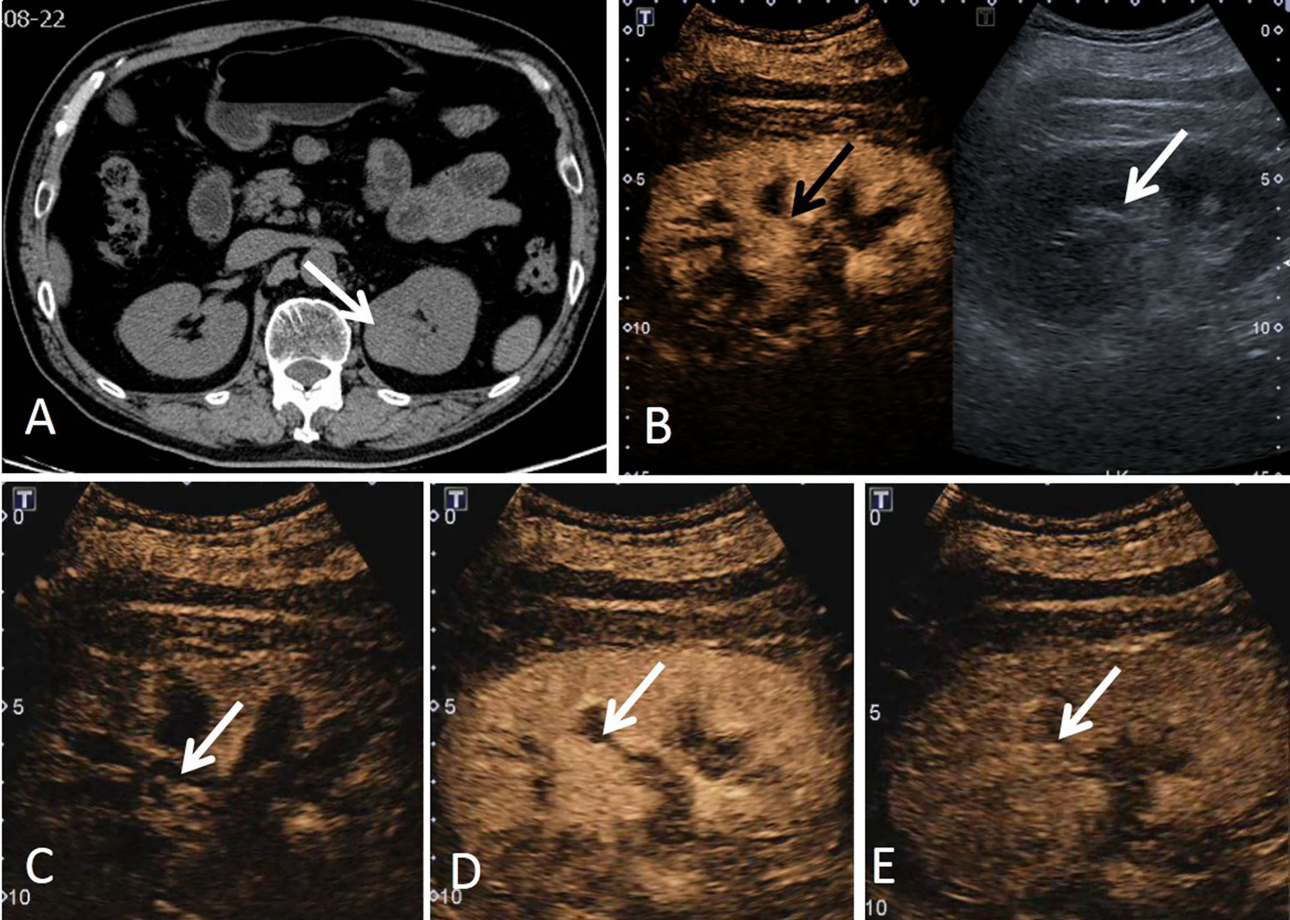
A 69-year-old man had a ccRCC in the left kidney with the size of 2.9 × 2.1 cm. **(A)** Non-enhanced CT scan showed a mass (white arrow) that was poorly demarcated from the renal pelvis and surrounding vessels. **(B)** According to the position displayed on CT, the simultaneous display mode of CUS (right) and CEUS (left) was performed, CEUS showed a suspicious mass with hyperenhancement (black arrow), but it was not clear on CUS (white arrow). **(C)** On CEUS imaging, the contrast agent entered the mass (white arrow) synchronously with the adjacent renal cortex. **(D)** The tumor showed homogeneous enhancement, and the enhancement intensity was slightly higher than that of the adjacent renal cortex (white arrow). **(E)** CEUS imaging showed that the tumor had a slow-out pattern (white arrow) compared to the renal cortex.

All the cases were performed in the simultaneous display mode of both CUS and CEUS images. Of the 10 cases with ccRCC, 7 cases (70.0%) were isoechoic, and the other 3 cases (30.0%) were hypoechoic on CUS. No renal pelvis separation was observed in the 10 cases of ccRCC. The largest ccRCC was not clearly demarcated from the renal pelvis and surrounding blood vessels on CT, and it was mistaken for a tumor of the renal pelvis (**Figure 4**); however, it was not observed on CUS. Of the six cases with UCRP, all the lesions were hypoechoic on CUS. Slight renal pelvis separation was observed in three cases (**Table 2**).

**Table 2:** CEUS and CUS characteristics of the 16 renal masses.

Case	Enhancement intensity	Homogeneity (CEUS)	Wash-in pattern	Wash-out pattern	Perilesional rim-like enhancement	Echogenicity	Homogeneity (CUS)	Blood flow signal
1	Hyperintense	Heterogeneous	Synchronous	Synchronous	Absent	Hypoechoic	Heterogeneous	Yes
2	Hyperintense	Heterogeneous	Synchronous	Fast	Absent	Isoechoic	Heterogeneous	Yes
3	Hyperintense	Heterogeneous	Slow	Synchronous	Absent	Isoechoic	Heterogeneous	No
4	Hyperintense	Homogeneous	Synchronous	Slow	Absent	Isoechoic	Homogeneous	Yes
5	Hyperintense	Homogeneous	Synchronous	Slow	Absent	Isoechoic	Homogeneous	Yes
6	Hyperintense	Homogeneous	Synchronous	Slow	Absent	Hypoechoic	Heterogeneous	No
7	Hyperintense	Heterogeneous	Synchronous	Synchronous	Absent	Isoechoic	Homogeneous	No
8	Hyperintense	Homogeneous	Synchronous	Fast	Absent	Isoechoic	Homogeneous	No
9	Hyperintense	Homogeneous	Fast	Synchronous	Absent	Hypoechoic	Homogeneous	No
10	Hyperintense	Homogeneous	Fast	Slow	Absent	Isoechoic	Homogeneous	No
11	Isointense	Homogeneous	Slow	Fast	Absent	Hypoechoic	Heterogeneous	No
12	Hypointense	Homogeneous	Slow	Fast	Absent	Hypoechoic	Heterogeneous	No
13	Hypointense	Homogeneous	Slow	Fast	Absent	Hypoechoic	Homogeneous	No
14	Isointense	Heterogeneous	Fast	Slow	Absent	Hypoechoic	Heterogeneous	No
15	Isointense	Heterogeneous	Slow	Fast	Absent	Hypoechoic	Heterogeneous	No
16	Isointense	Heterogeneous	Slow	Synchronous	Absent	Hypoechoic	Heterogeneous	No

CEUS, contrast-enhanced ultrasound; CUS, conventional ultrasound.

## Discussion

The European Association of Urology Guidelines on Renal Cell Carcinoma suggested that CECT and MRI were the preferred imaging modalities for the characterization and diagnosis of renal cell carcinoma (RCC), and CEUS can be used as a supplementary method for patients with chronic renal failure or known allergy to iodide or gadolinium-containing contrast agents (10). Among all the diagnosis methods, CEUS has the advantages of minimal invasiveness, no radiation, real time, and no burden on renal metabolism, which may be helpful for tumor diagnosis, especially for early differential diagnosis (1).

In recent years, with the development of medical imaging technology, more and more small renal tumors have been detected, and the sonographic characteristics have been summarized (11). However, for some tumors with atypical sonographic appearances, misdiagnosis and missed diagnosis may occur. Although great advances had been achieved in imaging techniques, the detection of small renal tumors remained a challenge for CUS. These lesions commonly showed blurred margins, which were confused with the surrounding renal cortex. Moreover, the sensitivity of CUS was fundamentally correlated to the size of the tumor. The smaller the tumor size, the more difficult it can be detected by CUS; however, CEUS was less affected by these factors.

Of the 16 patients in our study, 10 patients (62.5%) had ccRCCs. The incidence of ccRCC is 70% of renal carcinoma (12). Most of the ccRCCs are asymptomatic and detected by incidental radiological examination (13). In our series, seven cases were asymptomatic, and three cases had pain and discomfort in the waist. On CUS, seven (70.0%) cases were isoechoic, which were indistinguishable from the surrounding renal cortex, and the lesions did not protrude from the renal capsule either. The other three cases (30.0%) were hypoechoic on CUS, which was similar to the echogenicity of the renal cone nearby. Therefore, these lesions were easily missed on CUS. However, the 10 cases with ccRCCs showed high enhancement on CEUS, which was easily distinguished from the adjacent renal parenchyma. Li et al. also reported that 26.3% of small tumors could not be detected by CUS, while all the tumors could be distinguished from the adjacent renal cortex on CEUS, by showing a sharper margin and high enhancement (8).

In the past, many studies have focused on the value of CEUS in the differential diagnosis between benign and malignant renal neoplasms. In addition to high enhancement, heterogeneous enhancement and perilesional rim-like enhancement were also highly suggestive of ccRCC (1, 14–17). Perilesional rim-like enhancement may represent the tumor’s pseudocapsule which was caused by the compression of the adjacent normal parenchyma, leading to ischemia, necrosis, and then deposition of fibrous tissue (18). However, it was not observed in all the ccRCCs. The main reason might be that the mass size in our study was smaller (mean, 2.0 ± 0.4 cm) than that of previous studies (14, 19). Intratumoral hemorrhage, necrosis, and compression might not be obvious in small tumors. Because of this, the rate (40.0%) of heterogeneous enhancement in the present study was lower than that in previous studies (19, 20). In addition, most ccRCCs showed a synchronous-in pattern, and it was consistent with the results reported by Li et al. (8). This characteristic might be related to the pathologic features of RCC, which was characterized by numerous thin-walled vessels with a rich blood flow (8). Our results showed that CEUS was helpful in detecting tumors in renal parenchyma that were undetectable on CUS; however, only some features appeared on CEUS, which could be helpful in the differential diagnosis. Therefore, the final diagnosis still depends on pathology findings.

In this study, there were six cases with UCRP which were not detected by CUS. The urothelial tumor originating from the renal pelvis accounts for about 10%–15% of all renal neoplasms, mainly including urothelial carcinoma (90%), squamous cell carcinoma (9%), and mucinous adenocarcinoma (1%) (21). Urothelial carcinoma was usually seen in elderly men with the most common symptom of gross or microscopic hematuria (22). Some patients might also have low back pain; however, 10%–15% of patients may be asymptomatic (23). In this study, gross hematuria was observed in three patients, and back pain was observed in four patients.

The main components of the renal sinus, including the collecting duct, lymphatic channels, adipose tissue, fibrous tissue, and nerve fibers, contribute to the hyperechogenicity of renal sinus on CUS, which might easily cover up iso- or hypoechoic lesions (24). When the renal pelvis separation is eccentric in shape, only the lateral margins are visible, and when the renal pelvis separation is lentil-like in shape, the lesion can be missed. The malignant tumors of the renal pelvis may be detected by showing neoplastic angiogenesis; however, color or power Doppler has limited sensitivity in detecting small vessels and low-speed blood flow. CEUS has a high sensitivity in detecting microvasculature, and renal pelvis tumors show local contrast enhancement on CEUS, which can clearly depict the outline of the tumor; thus, a definite diagnosis can be obtained (25). In this study, six cases of UCRP were indistinguishable from the hypoechoic renal pelvis, but they showed isoenhancement or hypoenhancement on CEUS. Therefore, they can be recognized by CEUS because the renal pelvis was not enhanced.

Of the six cases with UCRP, five cases (83.3%) showed a slow-in pattern, and four cases (66.7%) showed a fast-out pattern on CEUS. These findings were consistent with a previous study. Xue et al. (26) reported that slow-in, fast-out, and hypoenhancement were associated with renal urothelial carcinoma, and the enhancement intensity at peak was lower than that of the renal parenchyma. Compared with the renal cortex, the contrast agent in the tumor discharges earlier and faster, making the edge of the tumor easy to identify. Therefore, the renal urothelial tumor might be detected more easily by CEUS.

Sometimes, blood clots in the collecting system are difficult to distinguish from the renal pelvis tumor by CUS due to a similar sonographic appearance; however, on CEUS, tumors could show slight enhancement, which might be distinguished from blood clots with no contrast enhancement. In the case of UCRP combined with renal pelvis stones in our study, CT showed that there may be blood clots in the renal pelvis. No abnormalities were observed on CUS; however, CEUS showed an isoenhancement zone, indicating that there might be a tumor rather than blood clots. Our results showed that CEUS was helpful in detecting tumors in the renal pelvis that were undetectable on CUS, and it was also useful in the differential diagnosis. If a suspicious mass is observed on CT, and CECT is not available, CEUS can be recommended. However, the value of CEUS and CECT in the differential diagnosis of renal pelvis lesions needs to be further compared with a large sample.

The study has some limitations. First, the retrospective nature of this study might lead to potential bias in data collection. Second, the sample size of this study was limited, and no statistical analysis was performed. A further study with a large sample size should be performed. Furthermore, because the kidneys are located in the posterior peritoneum, it may be difficult to demonstrate blood flow in renal masses on color Doppler.

In conclusion, CEUS may be helpful in the diagnosis and differential diagnosis of renal tumors which were not observed on CUS, and it could increase confidence in clinical decision-making. CEUS might be an alternative method for some patients when CECT or MRI cannot be performed.

## Data availability statement

The original contributions presented in the study are included in the article/supplementary material. Further inquiries can be directed to the corresponding author.

## Ethics statment

The studies involving human participants were reviewed and approved by the ethical committee of Ruijin Hospital LuWan Branch, Shanghai Jiaotong University School of Medicine. Written informed consent for participation was not required for this study in accordance with the national legislation and the institutional requirements.

## Author contributions

Study concept and design: WZ, WWZ, and LT. Acquisition of data: LT, JF, WL, NY, and BM. Resources: LT, JF, WL, NY, and BM. Analysis and interpretation of data: WZ, LT, and JL. Drafting of the manuscript: LT. Critical revision of the manuscript for important intellectual content: WZ and JL. Technical or material support: WZ, WWZ, and JL. Study supervision: WWZ. All authors read and approved the final manuscript.

## Conflict of interest

The authors declare that the research was conducted in the absence of any commercial or financial relationships that could be construed as a potential conflict of interest.

## Publisher’s note

All claims expressed in this article are solely those of the authors and do not necessarily represent those of their affiliated organizations, or those of the publisher, the editors and the reviewers. Any product that may be evaluated in this article, or claim that may be made by its manufacturer, is not guaranteed or endorsed by the publisher.
